# Editors' selection of papers from China's academic journals

**DOI:** 10.1093/nsr/nwt018

**Published:** 2013-12-19

**Authors:** Xiang Li

## PHYSICS

### New discoveries of the deposition patterns and cracking behavior of colloidal droplets

A group of Chinese physicists, led by Dr Duyang Zang, have found that adding polyethylene oxide (PEO) to colloidal droplets greatly reduces the ‘coffee ring’ effect by blocking outward capillary flow and enhancing Marangoni flow. Their *in situ* observations also found that PEO additives reduce the propagation velocity of cracks by a factor of 50. In their paper, the colleagues proposed a crack nucleation mechanism that may be responsible for the sluggish cracking behavior. [*Sci China-Phys Mech Astron*, 2013; **56**(9): 1712–8]

## MATHEMATICS

### Exponential sums over galois rings

Galois rings and exponential sums over them have become very important tools to construct good error-correcting codes, sequences and combinatorial designs over ring $\mathbb{Z}_{p^r}$. Gauss sums and Jacobi sums over Galois rings, as examples of general exponential sums have been investigated by, Jin Li et al. In their work, Jin Li et al. presented an explicit description on the Gauss sums and Jacobi sums over Galois ring *GR*(*p*^2^, *r*), and showed that the values of these sums can be reduced to over a finite field $\mathbb{F}_{p^r}$ for all non-trivial cases. [*Sci China-Math*], 2013; **56**(7): 1457–65]

## PLANT & ANIMAL SCIENCE

### The first organelle-specific pH map of the endomembrane system of *Arabidopsis* cells

Recent work published by Jinbo Shen et al. reports on the first ever organelle-specific pH map for the *Arabidopsis* endomembrane system, using two pH sensors (PEpHluorin and PRpHluorin) and the transient expression system of *Arabidopsis* protoplasts as tools. Organelles in the plant secretory pathway revealed a gradual acidification, from pH 7.1 in the endoplasmic reticulum to pH 5.2 in the vacuole. Such a unique map will serve as an important tool for future studies in understanding organelle-mediated protein trafficking and organelle function in plants. [*Mol Plant*, 2013; **6**(5): 1419–37]

**Figure 1:**
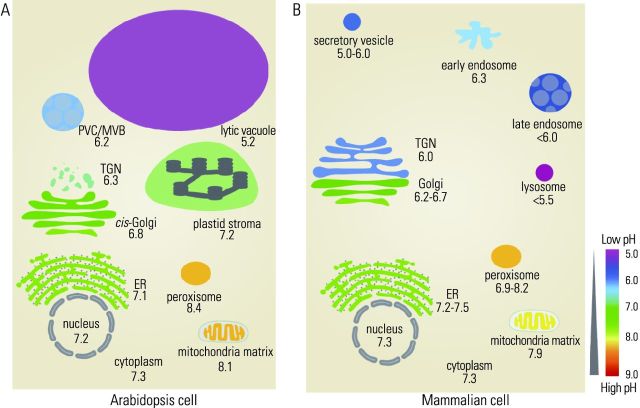
The pH map of intracellular organelles in *Arabidopsis* vs. mammalian cells. Published in *Mol Plant* 2013; **6**(5): 1419–37.

## BIOLOGY AND BIOCHEMISTRY

### Structural insights into receptor recognition of MERS Coronavirus

Recent research published by Wang et al. on the molecular binding between the Middle East respiratory syndrome coronavirus (MERS-CoV) spike protein and its human receptor DPP4 has significant implications for the further understanding the pathogenesis of MERS-CoV. The results, achieved using X-ray crystallography and biochemical methods, can guide the development of therapeutics and vaccines against infection. Structural comparisons also revealed similarities and differences in the spike receptor-binding domain between MERS-CoV and severe acute respiratory symptom coronavirus (SARS-CoV), which bind to different human receptors but share clinical and genetic features. [*Cell Res* 2013; **23**(8): 986–93]

## GEOSCIENCES

### Environmental conditions of Chinese deserts and sand fields controlled by regional climate and human activity

Based on 400 optically stimulated luminescence ages and over 100 depositional records, Huayu Lu et al. reconstructed the changes in Chinese deserts and sand fields during the Last Glacial Maximum (LGM, c 26 000–16 000 years before present) and the Holocene Optimum (HO, c 9 000–5 000 years before present), periods characterized by cold–dry and warm–wet climates respectively. Their results show that, compared to the geological distributions of deserts and sand fields, human activity has expanded the area of active sand dunes. [*Chin Sci Bull*, 2013; **58**(23): 2775–83]

## GEOSCIENCES

### Formation of Xiuyan impact crater in Northeast China

The Xiuyan Crater (40°21^′^55^′′^N, 123°27^′^34^′′^E) in the Liaoning Province, China, was listed in the Earth Impact Database in 2010. This well-preserved, simple bowl-shaped crater measures 1 800 m in diameter with a depth of about 150 m. Using crater impact morphological theory and mathematical modeling, scientists studied the cratering process and morphological features. The recently published work demonstrated that the Xiuyan Crater represents not just a tangible link between earth and space, but has important scientific applications, facilitating further studies into e.g. the regional paleo-climate and environment [*Sci China-Earth Sci*, 2013; **56**(10): 1629–38]

**Figure 2:**
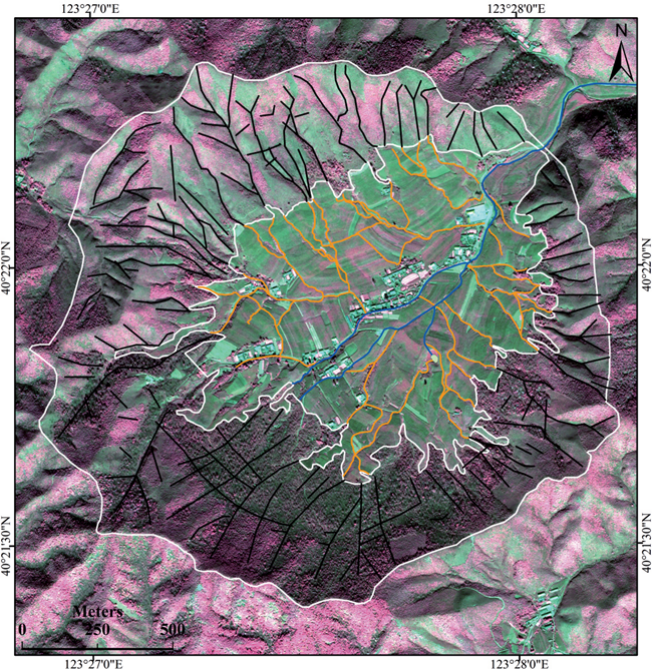
Spot 5 image shows the fractures and drainage system of Xiuyan Crater. White lines indicate inner and outer boundaries; black lines are crater wall fractures; yellow lines are gullies on the floor and blue are rivers. Published in *Sci China-Earth Sci* 2013; **56**(10): 1629–38.

## INFORMATION SCIENCE

### Over 50 scientists from China and Sweden collaborate to advance mobile communications

From 2009 to 2011, a strategic cooperation project between China and Sweden on the IMT-Advanced and beyond technologies saw over 50 scientists collaboratively investigating perspective emerging technologies. The research, which put a spotlight on wireless transmission, networking, and protocols, has been featured in a special issue of Future Wireless and Mobile Communications (published in February 2013). The issue introduces the joint research work and reports on the state-of-the-art mobile communication techniques, covering coordinated relay and MIMO transmission, cross-layer design of radio link and coding, and heterogeneous and self-organizing wireless networks. [*Sci China-Inf Sci*, 2013; **56**(2): 020300(1)]

